# Involuntary Psychiatric Admission: Arbitrary Deprivation of Liberty or a Human Right?

**DOI:** 10.3389/fpsyt.2022.879093

**Published:** 2022-07-11

**Authors:** Sergio Tamai

**Affiliations:** ^1^Department of Psychiatry, University of São Paulo, São Paulo, Brazil; ^2^Department of Psychiatry and Medical Psychology, Santa Casa São Paulo Medical School, São Paulo, Brazil

**Keywords:** ethics, involuntary admission, psychiatry, mental health, human rights

## Abstract

In 2008 Brazil ratified The United Nations (UN) Convention on the Rights of Persons with Disabilities (CRPD) an international legal instrument specifically tailored to stipulate the rights of persons with disabilities and include those with serious mental disorders. United Nations Committee set up to monitor the implementation of the Convention (CRPD Committee) lead to an insistence that involuntary detention and treatment of people with mental health (or “psychosocial”) disabilities are prohibited. There is a debate about this topic that poses an impossibility of involuntary psychiatric admission in hospital.

## Introduction

In 2008, Brazil ratified The UN Convention on the Rights of Persons with Disabilities 10 (CRPD) (1), an international legal instrument specifically tailored to stipulate the rights of persons with disabilities and include those with serious mental disorders. The United Nations Committee set-up to monitor the implementation of the Convention (CRPD Committee) leads to an insistence that involuntary detention and treatment of people with mental health (or “psychosocial”) disabilities are prohibited; this also applies to intellectual disabilities and degenerative conditions (e.g., dementia).

Convention on the Rights of Persons with Disabilities 10 reflects a change from pre-eminence of the Hippocratic principle of beneficence toward to patients' autonomy in decision about medical interventions. Autonomous decision-making, in its pure sense, is often difficult to achieve in psychiatric settings where a patient has a diminished capacity for autonomous decision-making and a potential risk of self-harm or harm to others. In this specific situation, restoration of the ability to make autonomous decisions is the end of the involuntary intervention. Beneficence becomes the primary ethical justification for involuntary admission in to a psychiatric hospital.

In patients suffering from a severe mental illness with clear impaired judgment capacity, challenging their autonomy decision in favor of hospitalization and treatment to benefit them is not a problem, but the same is not true when there is not a gross impact on individuals' reality testing as seen in personality disorders or substance abuse disorders.

Presently, a trend to a polarized view in all fields of society makes an adequate mental health policy difficult. Consequences of denying mental illness in favor of defending the individual's autonomy may be a failure to provide an adequate treatment to persons with mental illness and associated social marginalization and stigma (2).

In the United States, people with mental health disease illnesses are the majority of homeless population (3).

The latest estimates by the United States' Substance Abuse and Mental Health Services Administration reveal that a quarter of American homeless population is made up of individuals with mental disorders; although six percent of the general population suffers from mental illness (4).

There is an increasing trend of people mentally ill in prisons in the United State. Up to 25% of prison inmates are mentally ill (5).

According to Testa and West: “…*One reason that police cite as a motivating factor for taking people with mental disorders into criminal custody rather than to hospital emergency rooms is that the justice system is a more likely route through which long-term care can be achieved. It is unfortunate, but this is a direct result of the decreased average length of involuntary hospitalization* …” (2).

In 2001, Brazil has approved a legislation to state rights and ensure health assistance to persons with mental disorders: 10.216 Law. In this law, the principle of autonomy is present by patient's information and consent.

The ethical principal of beneficence justifies involuntary hospital admissions when patient's capacity to autonomous decision-making is compromised.

Article 6 of 10.216 Law defines three modalities of hospital admissions:

- Voluntary: with patient's informed consent.
- Involuntary: without the patient's informed consent and required by third party.
- Compulsory: determined by justice.

The informed consent model is adopted by 10.216 Law. In the informed consent, three conditions must be present:

1. the relevant information regarding treatment is adequately disclosed to the person.
2. the person is competent to consent.
3. the person can voluntarily consent.

If the person is considered not competethical justification for involuntary admission in to a psychiatric hospitalent to give an informed consent by the evaluating physician, the treatment decision must be made by a substitute decision-maker on behalf of the patient. A substitute decision-maker will typically be a (close) relative or a friend, ideally appointed by the patient herself; but if a social network is lacking, a legal guardian or alternatively the court can take up this role. Involuntary hospital admissions must be informed to a public attorney.

The 10.216 Law follows the principles adopted in most countries in the world, but its application is not always easy.

## Conclusion

The UN Convention on the Rights of Persons with Disabilities specifies how the principles of human dignity, equality, non-discrimination, autonomy, and social inclusion apply in the case of persons with disabilities. Such persons are taken to include those with serious mental disorders. It aims to ensure that such persons are treated on an equal basis with others, but an inflexible application of this statement in relation to involuntary hospital admissions may undermine the role of involuntary admission and treatment, which is to provide adequate mental health care to individuals whose mental disorders interfere with their rational ability to consent or refuse treatment.

## Author Contributions

The author confirms being the sole contributor of this work and has approved it for publication.

## Conflict of Interest

The author declares that the research was conducted in the absence of any commercial or financial relationships that could be construed as a potential conflict of interest.

## Publisher's Note

All claims expressed in this article are solely those of the authors and do not necessarily represent those of their affiliated organizations, or those of the publisher, the editors and the reviewers. Any product that may be evaluated in this article, or claim that may be made by its manufacturer, is not guaranteed or endorsed by the publisher.
